# A population model of the thalamo-cortical system during deep sleep

**DOI:** 10.1186/1471-2202-14-S1-P68

**Published:** 2013-07-08

**Authors:** Michael Schellenberger Costa, Arne Weigenand, Thomas Martinetz, Jens Christian Claussen

**Affiliations:** 1 University of Lübeck, Institute for Neuro- and Bioinformatics, Germany; 2 University of Lübeck, Graduate School for Computing in Medicine and Life Science, Germany

## 

Sleep has been shown to be crucial for the consolidation of memories, which can be enhanced by sensory or electric stimulation during slow wave sleep [1,2]. As the thalamo-cortical interaction is important for both the processing of sensory stimuli and the generation of slow waves a detailed understanding of its dynamical properties is needed, to

reveal the influence of one on the other. Population models have been used to investigate the behaviour of brain networks, may it be within specific regions [3] or between different structures of the brain [4]. Although they are able to reproduce a vast variety of oscillatory behavior of the awake brain [5], these models lack the hallmarks of a sleeping thalamus e.g. rebound bursts and waxing/waning of spindles. This is due to the fact that these models solely focus on the synaptic interaction between neural populations, while neglecting their intrinsic properties. However specific currents in the thalamic relay and reticular neurons are necessary for the generation of spindle activity and rebound bursts [6]. Therefore we adapt a cortical population model proposed by [7] to reflect the connectivity found within thalamic nuclei and extend it with the respective Hodgkin-Huxley type currents. We adjust the thalamic model to show the right oscillatory behaviour and investigate the interaction with the cortical region.
